# Dataset on analysis of dyeing property of natural dye from *Thespesia populnea* bark on different fabrics

**DOI:** 10.1016/j.dib.2017.11.063

**Published:** 2017-11-22

**Authors:** Kuchekar Mohini, Landge Tejashree, Navghare Vijay

**Affiliations:** a Pharmacognosy Department, P. E. Society's Modern College of Pharmacy, Nigdi, Pune, Maharashtra 411044, India; b Department of Pharmacology, School of Pharmacy, S.R.T.M. University, Nanded, Maharashtra, India

**Keywords:** Plant, Thespesia populnea, Bark, Natural dye, Fabrics

## Abstract

The natural dyes separated from plants are of gaining interest as substitutes for synthetic dyes in food and cosmetics*. Thespesia populnea (T. populnea)* is widely grown plant and used in the treatment of various diseases. This study was aimed to separate natural dye from *T. populnea* bark and analysis of its dyeing property on different fabrics. In this investigation pharmacognostic study was carried out. The pharmacognostic study includes morphological study, microscopical examination, proximate analysis along with the phytochemical study. The dyeing of different fabric was done with a natural dye extracted from *T. populnea* bark. The fabrics like cotton, butter crep, polymer, chiken, lone, ulene and tarakasa were dye with plant extract. The various evaluation parameters were studied. It includes effect of washing with water, effect of soap, effect of sunlight, effect of alum, effect of Cupric sulphate, microscopical study of fabrics and visual analysis of dyeing by common people were studied. In results, natural dye isolated from *T. populnea* bark could be used for dyeing fabrics with good fastness properties. The studies reveals that, the dyeing property of fabrics after washing with water and soap, exposed to sunlight does not get affected. It was observed that cotton and tarakasa stains better as compared with other fabrics. It was concluded that the ethanolic extract having good dyeing property.

**Specifications Table**

**Table:** 

**Subject area**	*Plant science*
**More specific subject area**	*Natural dyeing composites*
**Type of data**	*Text file, tables, photos and figures*
**How data was acquired**	*Experimental investigations*
**Data format**	*Calculated, Observations, analyzed, tabulated*
**Experimental factors**	*Dyeing of different fabrics by natural dye and evaluation of data*
**Experimental features**	*In the present study, dye was prepare and evaluated by using various parameters*
**Data source location**	*Department of Pharmacognosy, Modern College of Pharmacy, Pune, Maharashtra, India*
**Data accessibility**	*All data is given along with the article and will be accessible for education and research work.*

**Value of the data**

• Data provides method for how prepare the natural dye.
• This data is representing complete evaluation of dyeing of different fabrics and there evaluation.
• The data put here can be used as guideline to evaluate dyeing of fabrics
• This data may helpful for future development of natural dye from plant source.

## 1 Data

The crude drugs of natural origin such as plant, animal or marine source and there potent chemical constituents plays a significant role in various areas. The traditional remedies engross crude plant extracts containing large number of chemical constituents, in which specific chemical entity possesses high potency [1]. Natural dyes obtained from herbs are of budding concern as substitutes for artificial dyes in the pharmaceutical industry, textile and food [2]. Though several researchers reported the selection of plant as a raw materials and dyeing procedures, only minute information is available in literature.

*Thespesia populnea* (*T. populnea*) is a large tree (Family Malvaceae) found in tropical regions and coastal forests of India. The different parts of this plant are found to have useful therapeutic properties as well as used in various formulations [3]. The experimental findings revels the *T. populnea* commonly used in herbal medicine for various properties such as Dermatitis [4], Anti-oxidant activity [5], Alzheimers Disease [6], Antidiabetic activity [7], Synergistic activity [8], Immunomodulatory activity [9], Anti-inflammatory, analgesic and antipyretic [10], α-Amylase Inhibitory activity [11], antiulcer activity [12], Antioxidant and anti inflammatory [13] and Memory-enhancing activity [14].

*T. populnea* is added in formulation like Divya Stri Rasayan Vati, Guggul formula, Kamilari capsule, which is beneficial for digestive problems in females, It helps to give stability in mind as well as it is useful to cure the dark circles below the eyes in females. *T. populnea* contains Gossypol [15], Kaempferol, Quercetin, Kaempferol 3-glucoside, Quercetin 3-glucoside, rutin [16], Mansonones D, E and F [17], Nonacosane, lupenone, myricyl alcohol, lupeol, β-sitosterol and β-sitosterol-β-D-glucoside [18], Populneol [19], Thespesin [20], Thespesone and Thespone [21], The gossypol was isolated from the methanol extract of bark of *T. populnea* by using the mass triggered preparative HPLC [22].

The modern circumstance exhibit the request for plant drugs as a source of treatment for disease and cosmetics throughout the world. The extensive scientific impact and marketable prospective of traditional medicinal plants results in more and more international attention and global market demands [23]. In the present work, the dye extracted from bark of *T. populnea* in the field of textile. We carry out the study to explore the dyeing properties of *T. populnea* dye. The different evaluation parameters were examined during study like fabrics washing with water, fabrics washing with soap, effect of sunlight on treated fabrics, effect of alum, effect of cupric sulphate, microscopical study of fabrics, visual analysis of dyeing by common people. Dyeing conditions and fastness properties were investigated.

## 2 Experimental design, materials and methods

### 2.1 Plant material

**Fig. 1:**
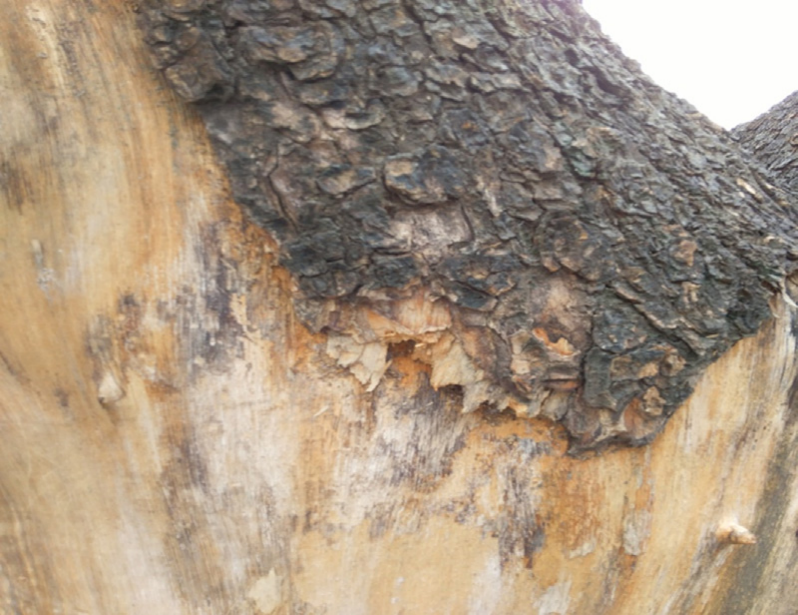
Bark of *T. populnea* plant.

Fresh bark of *T. populnea* grown-up at Dehuroad (Region of Pune), were collected on October and then dried under shade at room temperature and powder were made as shown in Fig. 1. The plant was authenticated by Botanical survey of India (BSI) with voucher specimen is preserved under reference number BSI/WRC/Tech./2013.

### 2.2 Fabric material

To study the dyeing properties of plant extract the different fabrics were procured from market at Pune. For the present study fabrics like cotton, butter crep, polymer, chiken, lone, ulene and tarakasa were selected.

### 2.3 Pharmacognostic study of plant

#### 2.3.1 Morphology of T. populnea bark

The various morphological parameters like colour, odour and taste height and width of collected bark sample were evaluated. The bark is odourless with fibrous fracture and brown in colour. It does not have any characteristic taste shown in Table 1.

**Table 1:** Morphological characters of *T. populnea* bark.

**Sr. No**	**Characteristics**	**Observations**
1	Colour	Brown
2	Odour	Characteristic
3	Taste	Pungent
4	Height	30 cm
5	Width	4 cm

#### 2.3.2 Microscopy of T. populnea bark

**Fig. 2:**
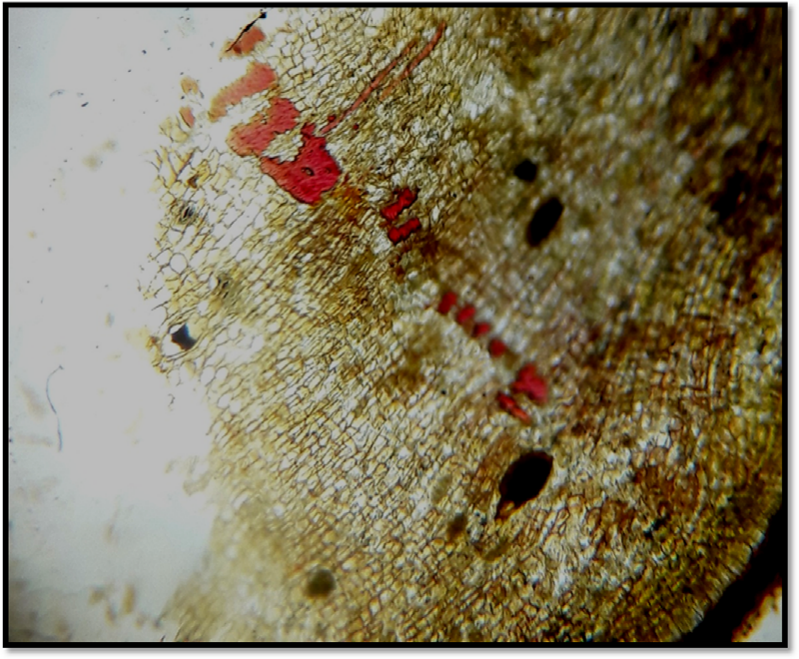
T.S. of *Thespesia populnea* bark.

The thin sections of bark were taken by normal section cutting method then mount on slide and observed under microscope. The various microscopical characters were observed in detail. The bark is flat to curved pieces. Due to numerous irregular scattered lenticels an outer surface was rough, fissured with irregular scales. The individual periderm bands present to the inner linining of outer bark. The periderm at outer part consists of phellem having deep fissures and phelloderm at inner narrow zone. Medullary rays and phloem fiber were seen shown in Fig. 2.

#### 2.3.3 Proximate analysis of plant

In this various parameters like ash values, extractive values, width of fiber, length of fiber, moisture content, swelling index foreign organic matter content and preliminary phytochemical evaluation were carried out [24] are shown in Table 2.

**Table 2:** Proximate chemical analysis.

**Sr. no**	**Physicochemical evaluation**	**Yield % w/w**	**Sr. no**	**Preliminary phytochemical evaluation**	**Observation**
1	Ash values	Yield %	3	Width of Fiber	36.48µ
	Total Ash	187%w/w	4	Length of fiber	3.91%
	Acid-insoluble ash	142%w/w	5	Foreign organic matter content	0.5
	Water-soluble ash	3.1	6	Moisture content	1.56%
2	Extractive values	Yield [% w/w]	7	Swelling index	0.5 ml
	Alcohol soluble	13.14% w/w			
	Water soluble	11 % w/w			

### 2.4 Preparation of plant extract

Initially the bark sample was air dried then subjected to make fine bark powder. This powder was extracted by using polar solvent like ethanol using soxhlet apparatus. The ethanolic extract of plant was in powder form. The final yield of ethanolic extract was found to be 13.14%.

In preliminary phytochemical evaluation carbohydrate, protein, amino acids, phenol, flavonoids and glycosides are present. The preliminary phytochemical evaluations are shown in Table 3.

**Table 3:** Qualitative chemical evaluation of extract. [24].

**Sr no**	**Test**	**Ethanol extract**
1	Alkaloids-	
	Mayer’s reagent	–
	Dragendorff’s reagent	–
	Hager’s reagent	–
	Wagner’s reagent	–
2	Carbohydrate-	
	Molisch reagent	+
	Fehling reagent	+
3	Steroid-	
	Liebermann Burchard reaction	–
4	Glycoside-	+
	Bortranger’s reaction	+
	Saponins	+
5	Tannins-	
	FeCl_3_ solution	+
	Lead acetate	+
6	Proteins-	
	Millon’s test	+
	Biuret test	+
7	Gums and Mucilage-	
	Alcoholic precipitation	–
	Molisch’s test	–

### 2.5 Dyeing with fabrics and its evaluations

**Fig. 3:**
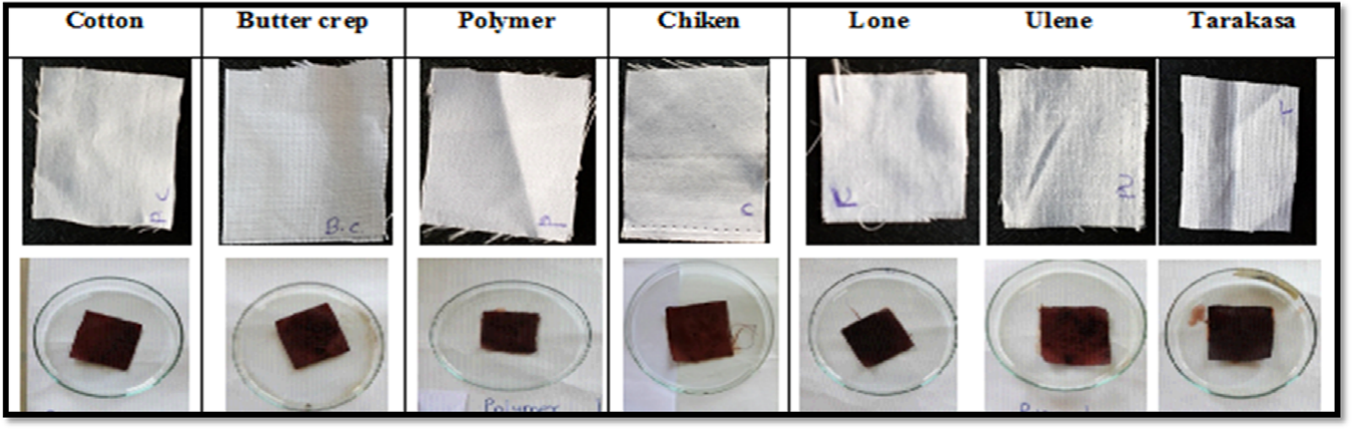
Application colour on fabrics.

All the fabrics were cut in to 10×10 cm size uniformly and transfer that cloth in the dye bath for 1 h for dyeing then dry it at room temperature shown in Fig. 3.

### 2.6 Evaluation of fabrics

#### 2.6.1 Fastness testing

The dyed fabrics were tested for fastness properties followed with standard methods, the particular tests were for colour fastness to washing with water ISO 105-E01:1989, colour fastness to washing with soap solution and colour fastness to light ISO 105-B02:1988.

**Fig. 4:**
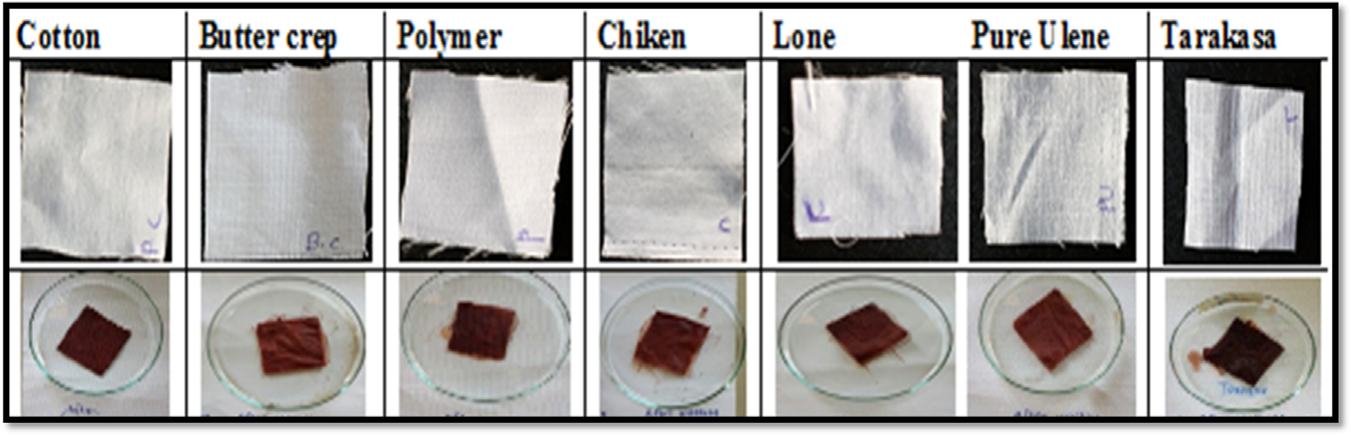
Fabrics after washing with water.
ii) *Effect of soap:* We have studied the effect of soap on colour consistency of treated sample. After washing with water the cotton, butter crep, polymer, chiken, lone, ulene and tarakasa fabrics subjected for soap treatment and then wash with water. The colour changes were observed. In this study, the effect of soap on treated cotton, butter crep, polymer, chiken, lone, ulene and tarakasa fabrics upon washing with soap solution shows that colour consistency of treated fabrics were same shown in Fig. 5.

i) *Effect of washing with water:* We have studied the effect of washing with water to study colour consistency of treated sample. After dyeing the cotton, butter crep, polymer, chiken, lone, ulene and tarakasa fabrics were wash with water to observe the colour change. In this study, the effect on treated cotton, butter crep, polymer, chiken, lone, ulene and tarakasa fabrics upon washing with water was shows that the colour consistency of treated fabrics were same shown in Fig. 4.

**Fig. 5:**
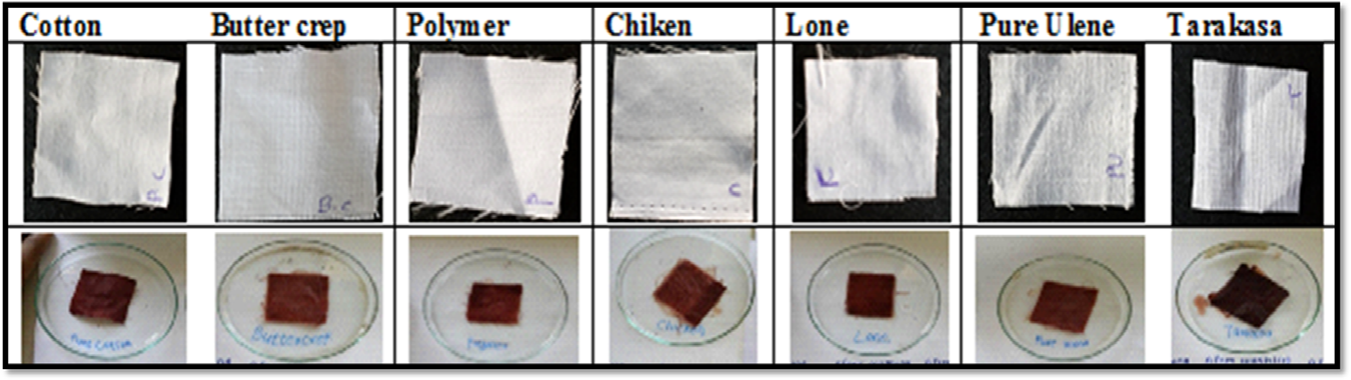
Fabrics after washing with soap.
iii) *Effect of sunlight:* The effects of direct sunlight on cotton, butter crep, polymer, chiken, lone, ulene and tarakasa fabrics were evaluated after washing with water and soap solution for 6 h and the color change was observed. There were no colour change of treated cotton, butter crep, polymer, chiken, lone, ulene and tarakasa fabrics when exposed to direct sunlight shown in Fig. 6.

**Fig. 6:**
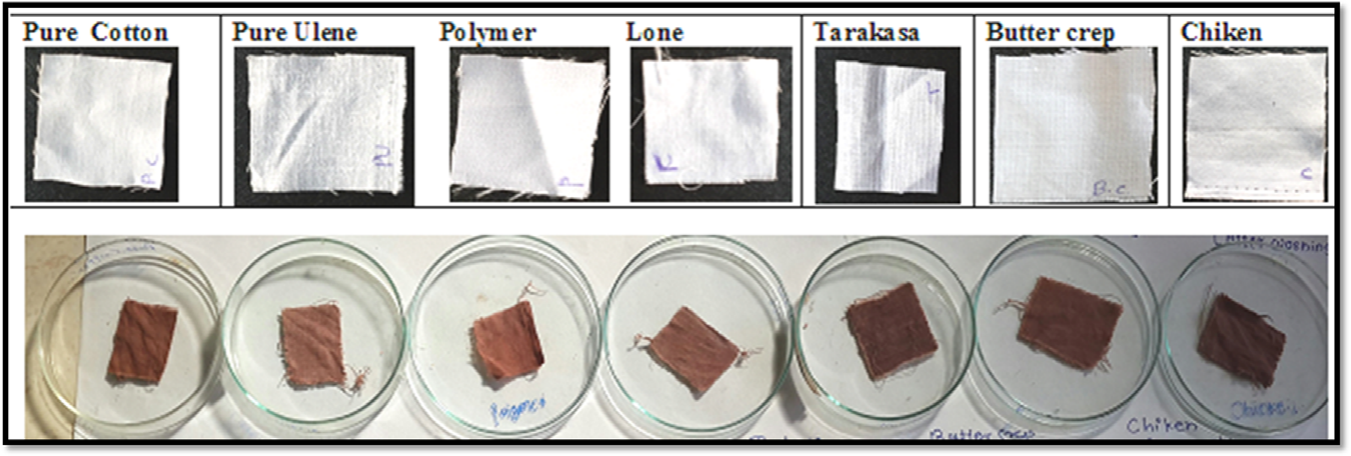
Effect of sunlight in fabrics.

#### 2.6.2 Effect of alum

**Fig. 7:**
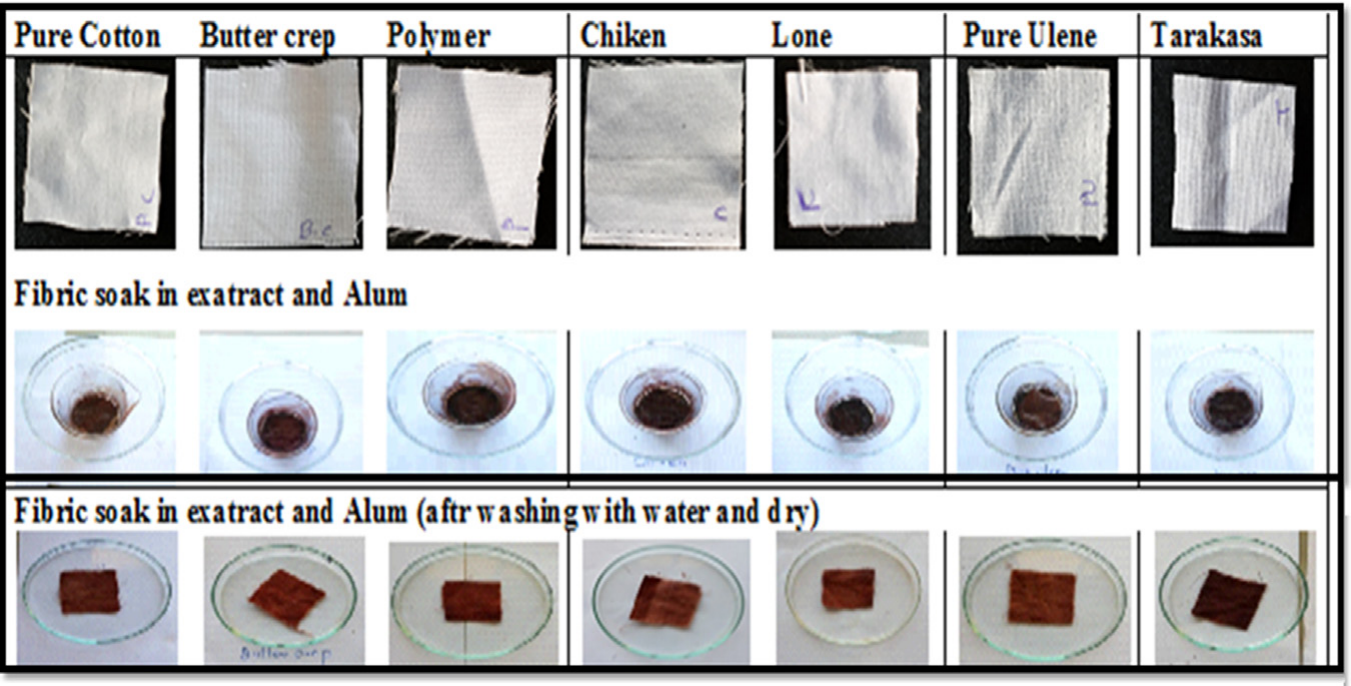
Treated fabrics soak in extract and Alum wash with water then dry. * Treated fabrics are first treated with extract and then subjected to treatment with alum.

**Fig. 8:**
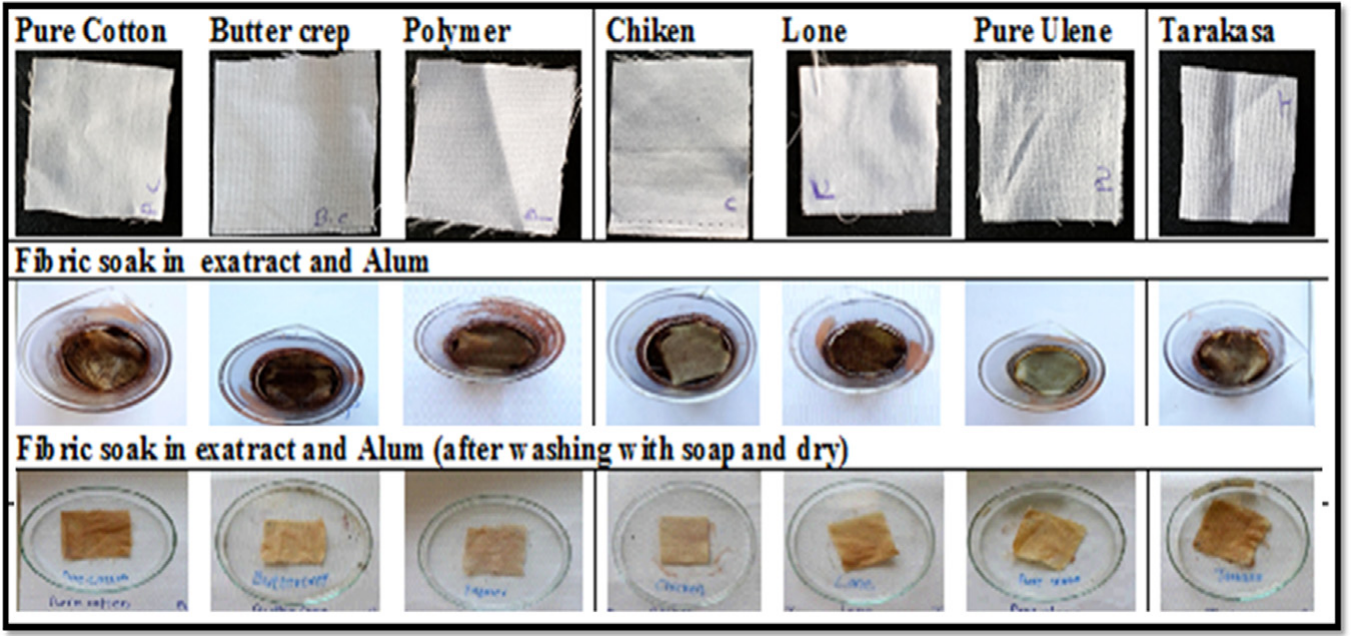
Normal fabrics soak in extract then in Alum wash with soap and then dry. * Normal fabrics those are directly put staining along with ethanolic extract and alum.

The comparative effect of normal and treated cotton, butter crep, polymer, chiken, lone, ulene and tarakasa fabrics with alum were studied. The comparative effect of normal and treated cotton, butter crep, polymer, chiken, lone, ulene and tarakasa fabrics with alum were studied. Normal fabrics those are directly put staining along with ethanolic extract and alum. Treated fabrics are first treated with extract and then subjected to treatment with alum. It was observed that treated fabrics showing good staining property and upon treatment with alum. It does not have colour consistency as that of normal fabrics even after washing with water and washing with soap shown in Fig. 7, Fig. 8 respectively.

#### 2.6.3 Effect of Cupric sulphate

**Fig. 9:**
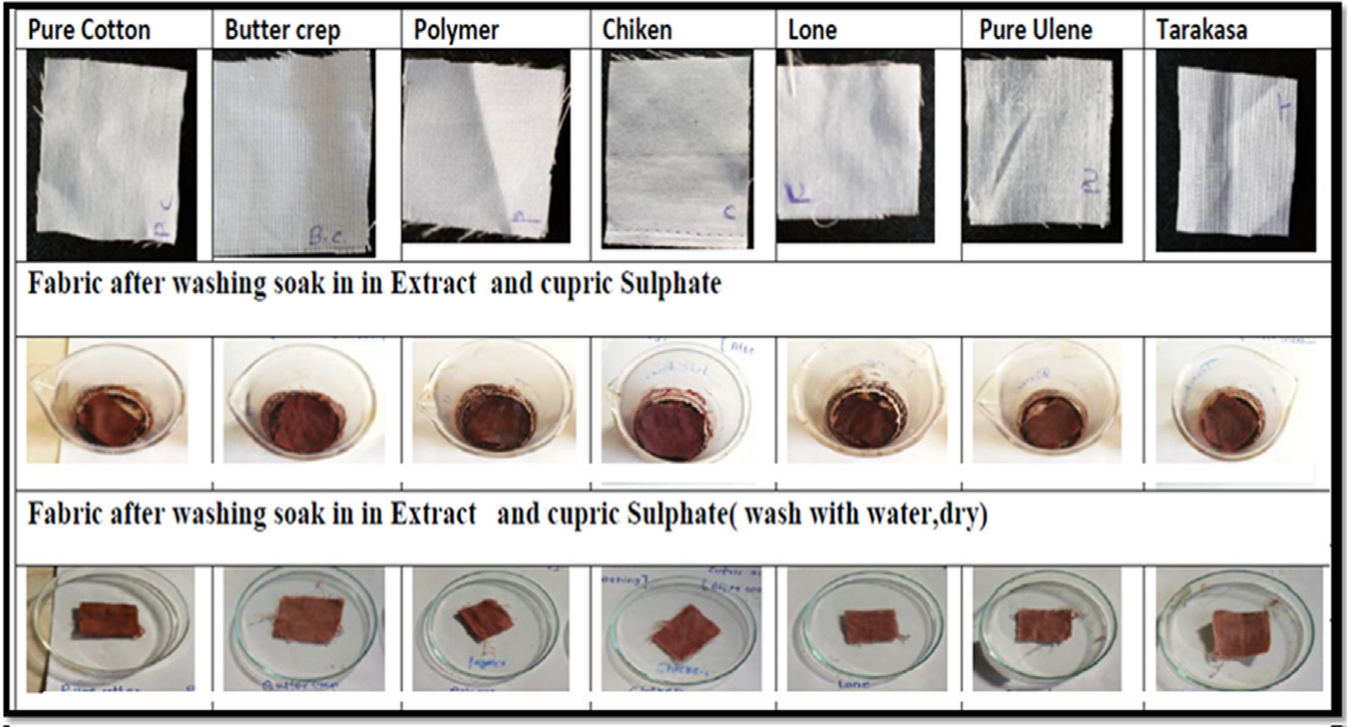
Treated fabrics soak in extract and cupric sulphate wash with water then dry. * Treated fabrics are first treated with extract and then subjected to treatment with Cupric sulphate.

**Fig. 10:**
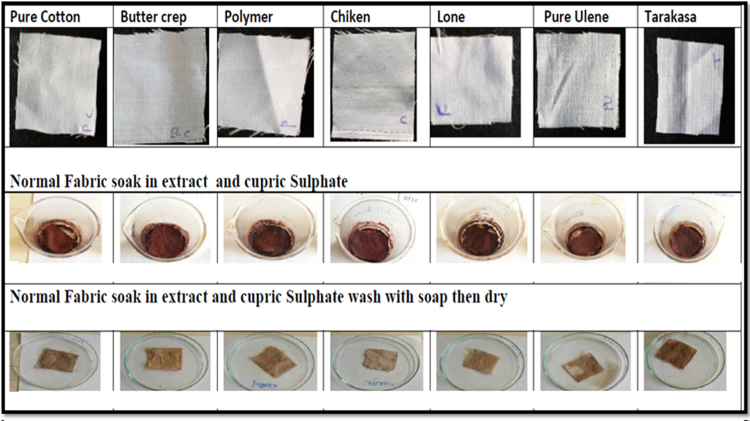
Normal fabrics soak in extract then in cupric sulphate wash with soap and then dry. * Normal fabrics those are directly put staining along with ethanolic extract and Cupric sulphate.

The comparative effect of normal and treated cotton, butter crep, polymer, chiken, lone, ulene and tarakasa fabrics with cupric sulphate were studied. The comparative effect of normal and treated cotton, butter crep, polymer, chiken, lone, ulene and tarakasa fabrics with Cupric sulphate were studied. Normal fabrics those are directly put staining along with ethanolic extract and Cupric sulphate. Treated fabrics are first treated with extract and then subjected to treatment with Cupric sulphate. It was observed that treated fabrics showing good staining property and upon treatment with Cupric sulphate. it slightly affect colour consistency but better than that of normal fabrics shown in Fig. 9, Fig. 10.

#### 2.6.4 Microscopical study of fabrics

**Fig. 11:**
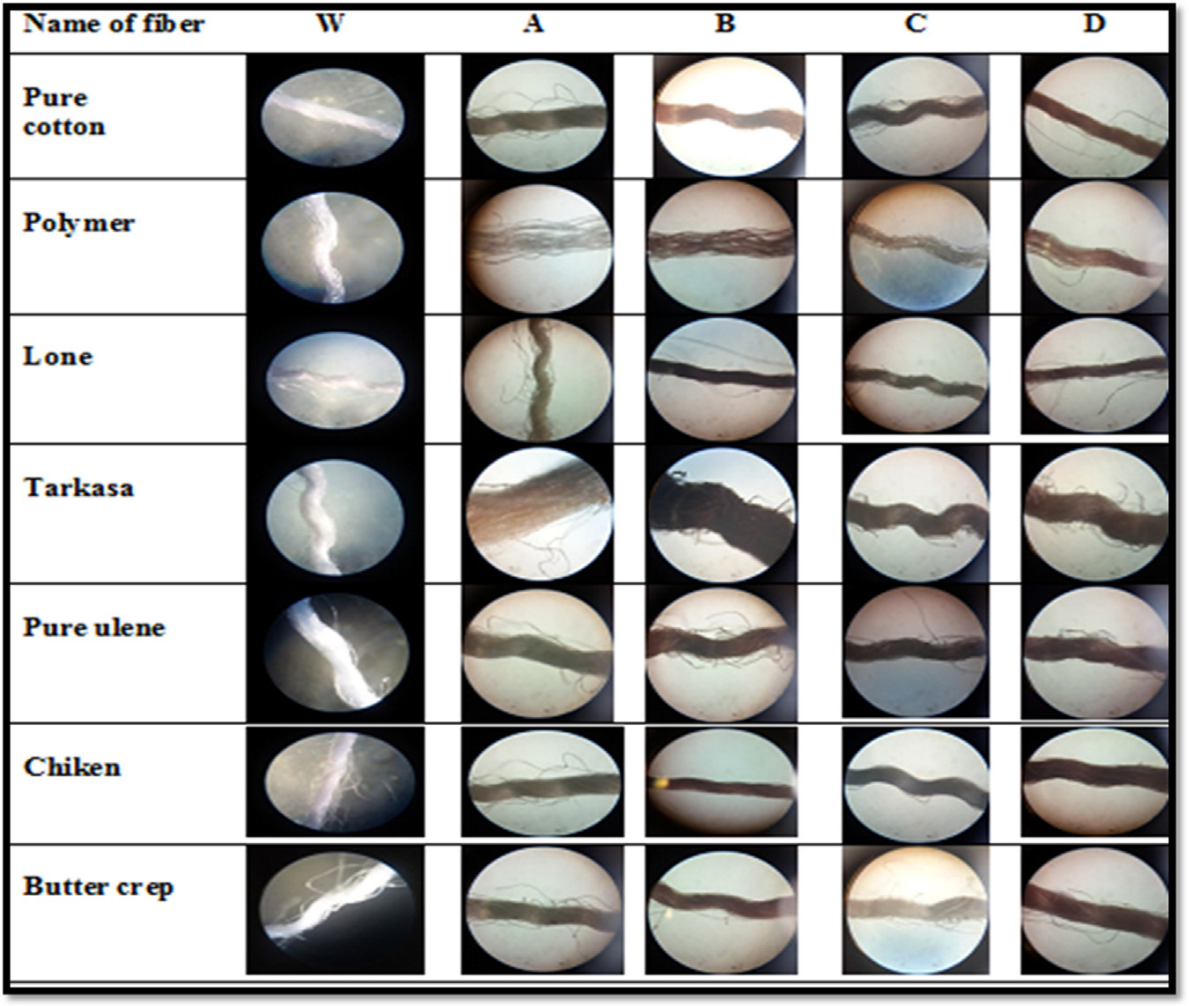
Microscopical study of fiber. W**.** Without dye fiber, A**.** Fiber soak in extract and Alum washing with water, B**.** Fiber soak in extract and Alum washing with soap, C**.** Fiber soak in extract and Cupric sulfate washing with water, D**.** Fiber soak in extract and Cupric sulfate washing with soap.

The cotton, butter crep, polymer, chiken, lone, ulene and tarakasa sample of fabrics were observed under microscope. All these fabrics were observed under microscope i.e. cotton, butter crep, polymer, chiken, lone, ulene and tarakasa shown in Fig. 11.

#### 2.6.5 Visual analysis of dyeing by common people

**Fig. 12:**
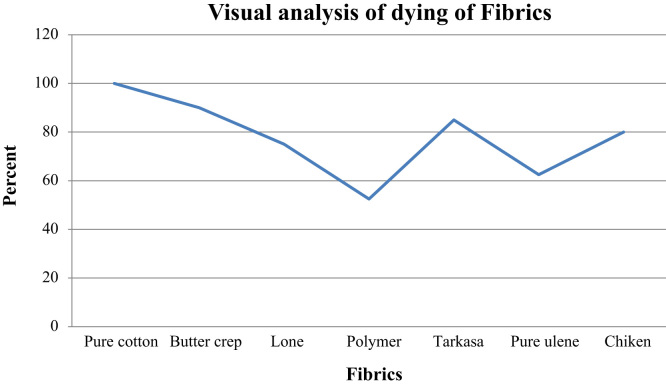
Visual analysis of dyeing by common people.

The cotton, butter crep, polymer, chiken, lone, ulene and tarakasa fabrics analyzed out by common people result are shown in Fig. 12.

Natural dye isolated from *T. populnea* bark could be used for dyeing fabrics with good fastness properties. It was found that the dyeing property of fabrics washing with water and soap, exposed to sunlight does not get much affected. When dyeing fabrics treated alum and Cupric sulphate colour is slightly changes with Cupric sulphate. So, it is concluded that alum will be the best mordent can be used.
